# Computational Analysis of Pumping Efficacy of a Left Ventricular Assist Device according to Cannulation Site in Heart Failure with Valvular Regurgitation

**DOI:** 10.1155/2016/6930482

**Published:** 2016-12-26

**Authors:** Aulia Khamas Heikhmakhtiar, Ki Moo Lim

**Affiliations:** Department of IT Convergence Engineering, Kumoh National Institute of Technology, Gumi-si, 39253, Republic of Korea

## Abstract

Mitral valve regurgitation (MR) causes blood to flow in two directions during contraction of the left ventricle (LV), that is, forward into the aorta and backward into the left atrium (LA). In aortic valve regurgitation (AR), leakage occurs from the aorta into the LV during diastole. Our objective is to analyze the contribution of a left ventricular assist device (LVAD) to MR and AR for the following two different cannulation sites: from the LA to the aorta (LAAO) and from the LV to the aorta (LVAO). Using a computational method, we simulated three ventricular conditions (normal [HF without valvular regurgitation], 5% MR, and 5% AR) in three groups (control [no LVAD], LAAO, and LVAO). The results showed that LVAD with LAAO cannulation is appropriate for recovery of the MR heart, and the LVAD with LVAO cannulation is appropriate for treating the AR heart.

## 1. Introduction

Worldwide, heart failure (HF) is the major cause of death, according to data from the American Heart Association [1]. HF is characterized by progressive inability of the heart to pump the appropriate amount of blood through the body [2]. In HF, the structure (from molecular level to organ level) and function of the heart are considerably altered, resulting in reduced cardiac performance. The heart becomes dilated and cardiac cells develop abnormal ion channel gates, leading to abnormal electrical activation, slow electrical conduction, and disarranged calcium activation, which causes ineffective contraction, dyssynchrony of depolarization, and myofiber shortening, among several other effects. Severe HF develops without prompt and appropriate treatment. HF can have several causes, such as an unhealthy lifestyle with inappropriate nutrition and/or drug use, smoking, and lack of exercise. Genetic or congenital diseases, birth defects, age-related changes, and infections can also result in HF [3]. One of the factors that can lead to severe HF is valvular regurgitation [4].

In valvular regurgitation, valve(s) do not close completely during the ejection and/or diastolic phase. This creates backflow of blood into the atrium and/or the ventricle, decreasing blood flow in the body [3, 5]. The most common type is mitral valve regurgitation (MR). A mitral valve leak can cause blood to flow in two directions during contraction of the left ventricle (LV), that is, forward into the aorta and backward into the left atrium (LA). This increases the pressure and volume in the LA, which leads to increased pressure in the pulmonary vein. Severe regurgitation can result in fluid accumulation in the lung [6]. In aortic valve regurgitation (AR), leakage from the aorta into the LV during diastole leads to increased pressure and volume in the LV. As a result, the heart works harder with each beat. This can cause thickening in the heart wall and eventually leads to HF [3]. AR is commonly caused by weakening of valve tissue owing to the aging process.

A left ventricular assist device (LVAD) can be used to support heart function as a bridge or as a treatment for patients with HF [7]. The LVAD increases cardiac output, unloads the ventricle, and improves coronary circulation [8, 9]. Timms et al. performed an experimental study of the ventricular unloading effect of an LVAD for the following two cannulation sites: from LV to ascending aorta (LVAO) and from LA to ascending aorta (LAAO) [10]. They concluded that LVAO is appropriate for a class IV patient who uses an LVAD as a bridge to transplantation or as a destination therapy. However, for a class III patient, alternative cannulations, such as LAAO and ascending aorta to descending aorta (AODA), are applied for recovery, as these cannulations do not alter the damaged ventricle invasively. The drawback of using LAAO or AODA is that it causes a serious problem in the long term, such as valve stenosis and blood thrombosis, owing to low ejection fraction (EF) [11]. Previously, using computational methods, we developed several cannulation methods for LVAD support as a bridge to recovery [12]. The cannulation sites were AODA, LAAO, and LVAO. The results showed that LAAO is more advantageous than AODA in terms of ventricular unloading and coronary perfusion.

The purpose of this study is to analyze the effect of an LVAD on AR and MR for different cannulation sites. Using a computational method similar to the numerical method used for developing previously reported cardiac electromechanical models [12, 13], we simulated a failing canine ventricular model under the following three conditions: HF without valvular regurgitation (normal), HF with 5% AR, and HF with 5% MR. We applied these conditions to the following three groups based on the LVAD cannulation site: control, LAAO, and LVAO. In the control group, the conditions (normal, AR, and MR) were modeled without the use of the LVAD. In the LAAO group, the LVAD was used for all conditions, with the cannulation site placed between the LA and aorta. In the LVAO group, the LVAD was used for all conditions, with the cannulation site placed between the LV and aorta

## 2. Materials and Methods

### 2.1. Mechanical Ventricular Method

**Figure 1:**
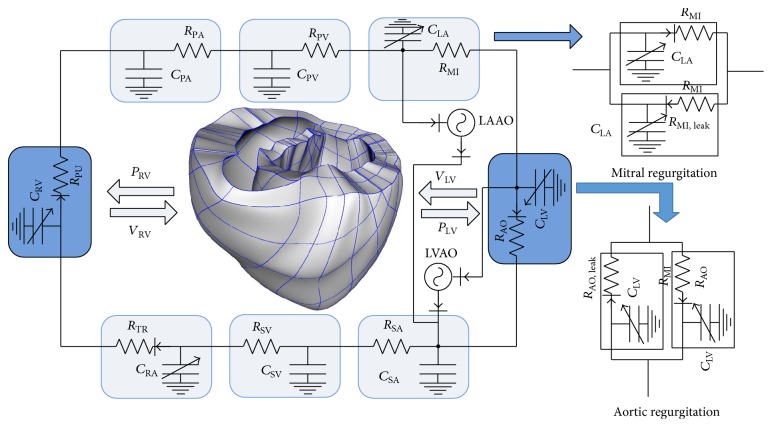
Schematic diagram of the finite-element ventricular electromechanical model coupled with the circulatory, valvular regurgitation, and LVAD models (LAAO and LVAO). *P*_RV_: RV pressure, *V*_RV_: RV volume, *P*_LV_: LV pressure, *V*_LV_: LV volume, *R*_PA_: pulmonary artery resistance, *C*_PA_: pulmonary artery compliance, *R*_PV_: pulmonary vein resistance, *C*_PV_: pulmonary vein compliance, *R*_MI_: mitral valve resistance, *C*_LA_: left atrium compliance, *R*_AO_: aortic valve resistance, *R*_SA_: systemic artery resistance, *C*_SA_: systemic artery compliance, *R*_SV_: systemic vein resistance, *C*_SV_: systemic vein compliance, *R*_TR_: tricuspid valve resistance, *C*_RA_: right atrium compliance, and *R*_PU_: pulmonary valve resistance.

We used a previously developed canine electromechanical model based on magnetic resonance imaging [14, 15]. Then, we combined a three-dimensional (3D) image-based failing canine ventricle with a lumped model of the circulatory system and an LVAD model, as shown in Figure 1. The LVAD was modeled as a flow generator. The flux of the LVAD was set to have a constant value of 40 mL/s. For the simulation protocol, we used electromechanical coupling model. The electromechanical model consisted of two compartments, that is, electrical and mechanical. Each compartment was coupled using Ca^2+^ activation. For electrical simulation, we implemented a sinus rhythm by applying the electrical activation time proposed by Durrer et al. [16] with a basic cycle length of 600. We obtained intracellular Ca concentration profiles at all computation nodes from the electrical simulation. Then, these profiles were applied to the mechanical simulation as input parameters. For the mechanical simulation, we performed simulation until 20 seconds, until a steady state response was obtained. The myocardial filament model developed by Rice et al. [17] was used for the mechanical compartment. To mimic the HF model, the passive scale factor of the strain energy function was increased by five times [18], and the normal myocardial cell calcium transient was reduced by 70% [19]. The schematic diagram in Figure 1 shows the combination of LAAO and LVAO in the model; however, we simulated each condition separately. The schematic diagram represents the blood circulatory system in the human body. It consists of a 3D ventricular diagram and a nondimensional lumped model. Each compartment of the system exchanges information about the pressure and volume in the LV and right ventricle (RV). *P*, *V*, *C*, and *R* denote pressure, volume, capacitance, and resistance, respectively.

We analyzed several mechanical responses such as 3D adenosine triphosphate (ATP) consumption rate distribution, average ATP consumption rate, pressure waves of the LA, LV, and systemic artery, LV pressure and volume, and LV stroke work. The ATP consumption rate was calculated by integrating the ATP consumption of each node, which represents the myofilament model in one cycle proposed by Rice et al. [17]. In the single myofilament model, the ATP consumption rate (*E*) per unit volume is the product of cross-bridge detachment rate (*g*_xbT_) and the single overlap fraction of thick filaments (SOVF_Thick_):

(1) $$\begin{array}{c} E=g_{xbT}\times {SOVF}_{Thick}. \end{array}$$

### 2.2. Valvular Regurgitation Model

To model AR and MR, we added two additional branches parallel to the compartments of the LA and systemic arteries, which contained flow resistances and one-way valves in the lumped model of the circulatory system. *R*_MI,Leak_ and *R*_AO,Leak_ represent backward flow resistances through the mitral and aortic valves, respectively, in Figure 1. The flow mechanics of AR and MR are represented by the following equations:

(2) QMI=PLA−PLVRMIwhen  PLA>PLVPLA−PLVRMI,Leak=PLA−PLVRMI×SF100when  PLA≤PLV,

(3) QAO=PLV−PAORAOwhen  PLV>PAOPLV−PAORAO,Leak=PLV−PAORAO×SF100when  PLV≤PAO.

Equation (2) represents the flux direction of the mitral valve for two different conditions. The first condition represents the LA contraction phase, in which the LA pressure is higher than the LV pressure. The flux direction is from the LA to the LV. The second condition represents the LV contraction phase, in which the LV pressure is higher than the LA pressure. In this condition, the flux direction is from the LV to the aorta and LA. The flux flows back to the LA owing to 5% leak in the mitral valve. SF is the scale factor of the leakage from the valve. SF was set as 5 to represent 5% leak in the mitral valve. Equation (3) represents the leak in the aortic valve. *Q*, *P*, and *R* denote flow rate (mL/min), pressure (mmHg), and resistance (mmHg min/L), respectively, and subscripts MI, AO, LV, LA, and Leak denote mitral valve, aortic valve, left ventricle, left atrium, and regurgitation, respectively.

## 3. Results

**Figure 2:**
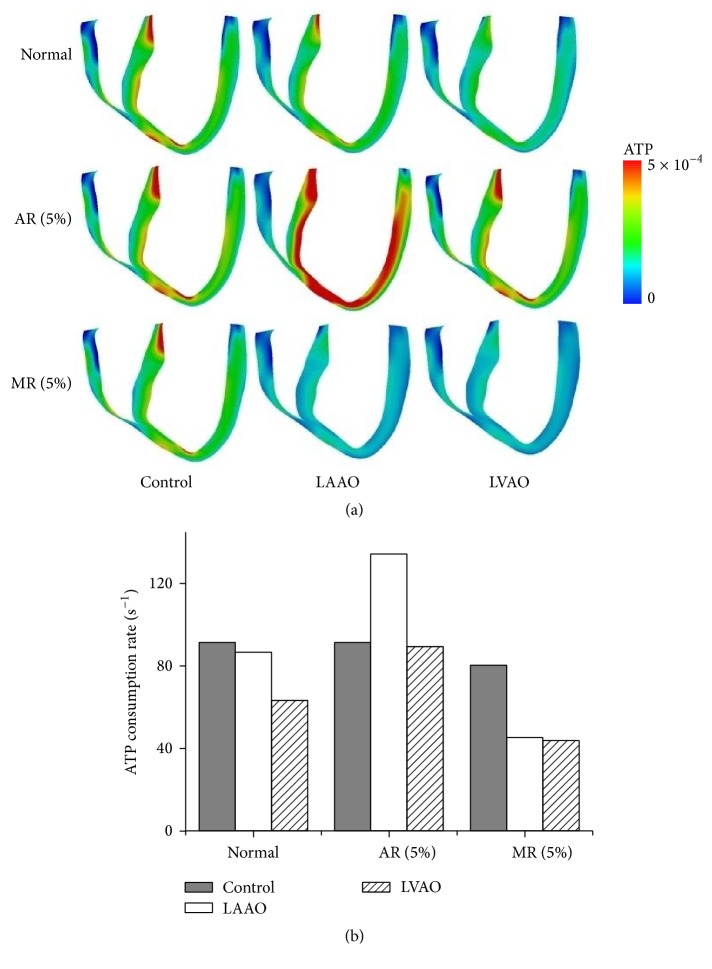
ATP consumption under normal, AR, and MR conditions for all groups.

Figure 2 shows the ATP contour distribution (Figure 2(a)) and the average ATP consumption rate (Figure 2(b)) for the normal (HF without valvular regurgitation), AR, and MR conditions for all groups. Under the normal condition, the LAAO and LVAO groups consumed 4.4% and 31% less ATP, respectively, as compared to the control group. The AR heart of the control group consumed 91 s^−1^ ATP. The AR heart with the LAAO cannulation site consumed 47% more ATP, and the AR heart with the LVAO cannulation site consumed slightly less ATP, as compared to the AR heart of the control group. The MR heart of the control group consumed 80 s^−1^ ATP. The MR heart consumed 45% less ATP with LVAD with the LAAO and LVAO cannulation sites, as compared to the control group. The LVAD with LVAO cannulation reduced ATP consumption for all heart conditions.

**Figure 3:**
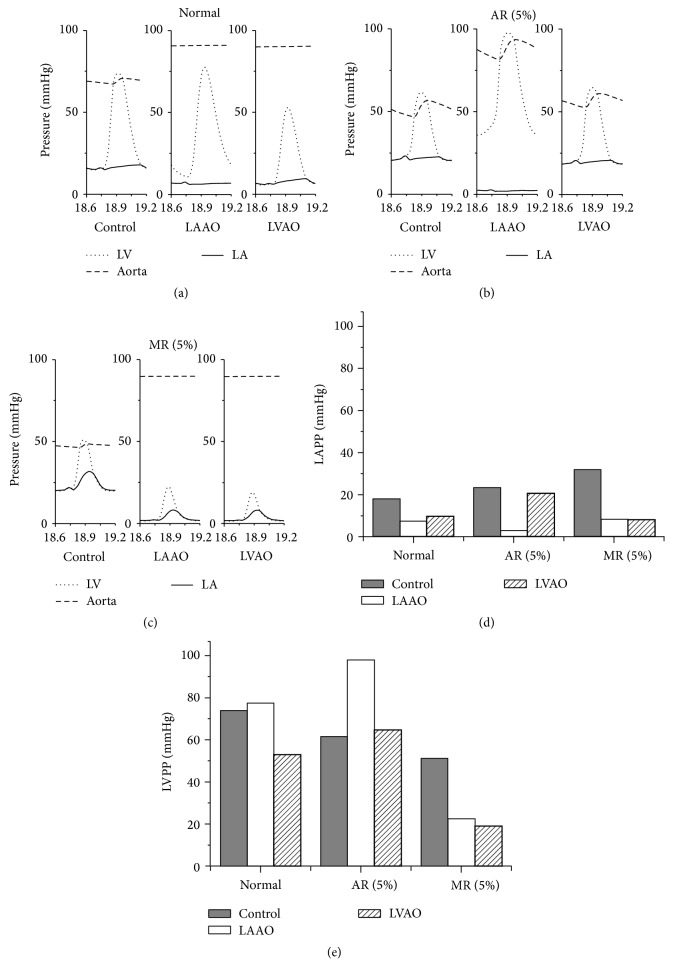
(a) LA, (b) LV, and (c) aortic pressures; (d) LAPP; and (e) LVPP under all conditions for all groups.

Figure 3 shows a comparison of the LA, LV, and aortic pressures for the normal, AR, and MR conditions in terms of the waveform (Figures 3(a)–3(c)), LA peak pressure (LAPP) (Figure 3(d)), and LV peak pressure (LVPP) (Figure 3(e)). A comparison between the mechanical responses for the normal, AR, and MR conditions was obtained from one cycle in steady state, which was from 18.6 seconds to 19.2 seconds. In Figure 3(a), the aortic pressure for the normal condition increased by up to 30% using the LVAD with both cannulation sites (LAAO and LVAO). This is due to the continuous flux pumped by the LVAD, which distributes blood continuously to the outlet of the cannulation (aorta). The peak pressure of the inlet chamber decreased by 44% and 25% for the LA with LAAO and LV with LVAO, respectively. In addition, the LV pressure for the normal condition with LAAO cannulation did not exceed the aortic pressure, which created isovolumic contraction and trapped the blood in the LV. This condition was also observed in the experimental study on severe HF by Timms et al. They showed that the use of LAAO in severe HF trapped blood in the LV during systole [10]. This trapped blood is the primary cause of blood thrombosis [11].

Figure 3(b) shows the LV, LA, and aortic pressures for the AR condition. The LV pressure for the control group under this condition was 16% lower compared to that under the normal condition. This is due to the leak from the aortic valve that reduces systolic efficacy. In addition, the LA pressure for the control group increased by 20% compared to that under the normal condition. The LVAD with LAAO cannulation increased the LV pressure for the control group by 34% under the AR condition, as compared to that under the normal condition. Increase in the LV and aortic pressures under the AR condition with LAAO cannulation was due to increase in the volume of the LV. The LAAO cannulation absorbed blood from the LA and sent it to the aorta. However, blood leaked to the LV from the aortic valve, which increased the LV volume. In addition, the LA pressure decreased owing to the unloading from LAAO cannulation. On the other hand, the use of LVAO cannulation did not affect the AR condition significantly. Even though the LVAD distributed the load to the aorta, the pressure of the systemic artery/aorta for coronary perfusion could not be maintained because of the leak.

In Figure 3(c), the LA pressure for the control group under the MR condition increased during the systolic phase owing to the mitral valve leak. Furthermore, the LV and aorta pressures could not be maintained. The use of the LVAD with LAAO and LVAO cannulations increased the systemic artery pressure, which resulted in high perfusion in coronary arteries. Additionally, both cannulations succeeded in unloading the blood from each chamber (LA for LAAO and LV for LVAO).

Figures 3(d) and 3(e) show the comparison between the LAPP and LVPP for all conditions and groups. It can be observed that the LAPP for the control group increased by 28% and 72% under the AR and MR conditions, respectively. On the other hand, the LVPP for the control group under the AR and MR conditions decreased by 16% and 31%, respectively.

**Figure 4:**
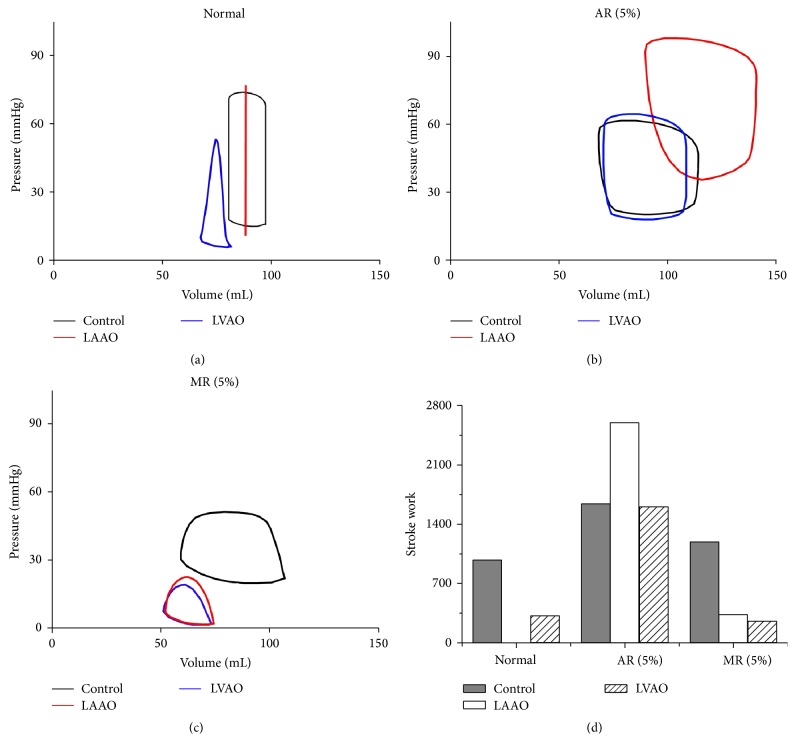
(a, b, c) Pressure-volume loops and (d) stroke work for all conditions.

Figure 4 shows the pressure-volume (PV) loop diagram for the normal (Figure 4(a)), 5% AR (Figure 4(b)), and 5% MR (Figure 4(c)) conditions, and the stroke work (Figure 4(d)) for all conditions. In Figure 4(a), the stroke volume of the control group under the normal condition was 20 mL, which implies it has 20% EF. The peak pressure was lower than that for the normal heart condition, and the end diastolic volume was higher than that for the normal condition. The normal condition of the control group was categorized as severe HF. Isovolumic contraction occurred under LVAD treatment with the LAAO cannulation site in the normal condition because of direct cannulation from the LA to the aorta, which completely distributed the blood, resulting in no change in the LV volume. The blood that remained in the LV could not flow through the aortic valve because the LV pressure under this condition is less than the aortic pressure. Thus, the blood was trapped in the LV.

In Figure 4(b), the LV under the AR condition exhibits a higher pressure and volume loop compared to the normal condition under control group when the LVAD with LAAO cannulation is implemented because of the leak in the aortic valve, which increases the volume and pressure of the LV. On the other hand, the PV loop for the AR condition is restored by implementing the LVAD with LVAO cannulation.

Figure 4(c) shows that the LVAD with LAAO and LVAO cannulation sites succeeded in unloading the blood and reduced the stroke work under the MR condition (Figure 4(d)). This implies that both cannulation sites distributed the blood appropriately and maintained the aortic pressure to increase coronary perfusion. Hence, LAAO or LVAO cannulation is suitable for treating a patient suffering from MR. However, as LAAO does not alter the LV structure and anatomy, we propose that LAAO cannulation is the most suitable for cardiac recovery of the MR heart. Stroke work exhibits similar pattern for the ATP consumption rate, except for the LAAO cannulation site under the normal condition. The stroke work for this condition was zero owing to 0% EF.

## 4. Discussion

We simulated a realistic 3D failing canine heart model under normal (HF without valvular regurgitation), 5% AR, and 5% MR conditions with two cannulation sites for an LVAD, that is, LAAO and LVAO. We analyzed mechanical responses such as ATP contour distribution, average ATP consumption rates, pressures of LV, LA, and aorta, PV loop diagram, and stroke work. The difference between the ATP consumption rates for the control groups was insignificant for the normal, 5% AR, and 5% MR conditions. The LVAD with LAAO and LVAO cannulations reduced the ATP consumption by 45% under the MR condition. Even though LAAO reduced the ATP consumption under the MR condition, it increased the ATP consumption by up to 47% under the AR condition, as compared to that for the control group under the AR condition.

In terms of the LV PV loop, isovolumic contraction was observed under the normal condition for the LVAD with LAAO cannulation. LAAO worsened pumping efficacy, in which the heart consumed considerable energy and the EF was zero. Even though the blood circulates with the assistance of the LVAD, the blood trapped in the LV causes thrombosis. In addition, LAAO cannulation under the AR condition is not applicable based on the simulation result because it increased the ATP consumption rate, LV pressure, and stroke work above normal values under the AR condition. In contrast, the LVAD with LVAO cannulation is more appropriate for treating an AR patient because it restores the ATP consumption and maintains the LV PV loop.

In the MR heart, the LVAD with LAAO and LVAO cannulation sites succeeded in restoring cardiac functions, which reduced ATP consumption, increased the aortic pressure for coronary perfusion, and provided sufficient cardiac output to the body. Nonetheless, the LVAD with LAAO cannulation is more appropriate for treating the MR heart. This option is suitable for a patient who uses LVAD solely for recovery and not as a bridge to transplantation or as a destination therapy. This is because LAAO cannulation does not alter the diseased LV directly, which is necessary for restoring cardiac functions.

## 5. Conclusions

The effect of the LVAD with LAAO and LVAO cannulation sites was predicted theoretically under the normal (HF without valvular regurgitation), 5% AR, and 5% MR heart conditions. The results showed that the LVAD with LVAO cannulation restored the hemodynamics of the heart under the AR and MR conditions. For the LVAD with LAAO cannulation, even though this cannulation restored the cardiac hemodynamics under the MR condition, it worsened the cardiac hemodynamics under the AR condition by increasing the ATP consumption, LV pressure/hypertension, and LV stroke work. In conclusion, the LVAD with LAAO cannulation is appropriate for recovery of the MR heart, and the LVAD with LVAO cannulation is appropriate for treating the AR heart.
